# Correction: The intracellular pathogen *Francisella* escapes from adaptive immunity by metabolic adaptation

**DOI:** 10.26508/lsa.202201733

**Published:** 2022-10-10

**Authors:** Kensuke Shibata, Takashi Shimizu, Mashio Nakahara, Emi Ito, Francois Legoux, Shotaro Fujii, Yuka Yamada, Makoto Furutani-Seiki, Olivier Lantz, Sho Yamasaki, Masahisa Watarai, Mutsunori Shirai

**Affiliations:** 1 Department of Microbiology and Immunology, Graduate School of Medicine, Yamaguchi University, Yamaguchi, Japan; 2 Department of Molecular Immunology, Research Institute for Microbial Diseases, Osaka University, Osaka, Japan; 3 Department of Ophthalmology, Department of Ocular Pathology and Imaging Science, Graduate School of Medical Sciences, Kyushu University, Fukuoka, Japan; 4 Joint Faculty of Veterinary Medicine, Laboratory of Veterinary Public Health, Yamaguchi University, Yamaguchi, Japan; 5 INSERM U932, PSL University, Institut Curie, Paris, France; 6 Systems Biochemistry in Pathology and Regeneration, Graduate School of Medicine, Yamaguchi University, Ube, Japan; 7 INSERM U932, PSL University, Laboratoire d’Immunologie Clinique, Centre d’Investigation Clinique en Biothérapie, Institut Curie (CIC-BT1428), Paris, France; 8 Laboratory of Molecular Immunology, Immunology Frontier Research Center, Osaka University, Osaka, Japan; 9 Division of Molecular Design, Medical Institute of Bioregulation, Kyushu University, Fukuoka, Japan; 10 Division of Molecular Immunology, Medical Mycology Research Center, Chiba University, Chiba, Japan

## Abstract

This study shows that this metabolic adaptation allows the intracellular bacterial pathogen *Francisella tularensis* to escape recognition by the host adaptive immunity.

We would like to apologize for an incorrect description in our manuscript entitled “The intracellular pathogen *Francisella tularensis* escapes from adaptive immunity by metabolic adaptation” (PMID: 35667686, PMCID: PMC9170078, DOI: 10.26508/lsa.202201441). We recently found that, during the revision process, a sentence of the results section in the published manuscript was mistakenly inserted. We think that the deletion does not affect the conclusion of the manuscript.

In the Results section

Where it reads:

Four of the substitutions are located in the zinc-binding region of the cytidine and deoxycytidylate deaminase domain and one substitution is in the bacterial bifunctional deaminase-reductase domain (InterPro database, https://www.ebi.ac.uk/interpro/) (Fig 2C). No substitutions were found in ribAB, ribH and ribC. Among the five substitutions in ribD, H80C and Q254R were shared with seven other virulent strains, based on genome sequences deposited in the KEGG database (Fig 2D).

It should read:

Four of the substitutions are located in the zinc-binding region of the cytidine and deoxycytidylate deaminase domain and one substitution is in the bacterial bifunctional deaminase-reductase domain (InterPro database, https://www.ebi.ac.uk/interpro/) (Fig 2C). Among the five substitutions in ribD, H80C and Q254R were shared with seven other virulent strains, based on genome sequences deposited in the KEGG database (Fig 2D).

